# Semi-automating abstract screening with a natural language model pretrained on biomedical literature

**DOI:** 10.1186/s13643-023-02353-8

**Published:** 2023-09-23

**Authors:** Sheryl Hui-Xian Ng, Kiok Liang Teow, Gary Yee Ang, Woan Shin Tan, Allyn Hum

**Affiliations:** 1 Health Services and Outcomes Research, National Healthcare Group, 3 Fusionopolis Link, #03-08, Singapore, 138543 Singapore; 2 Department of Palliative Medicine, Tan Tock Seng Hospital, 11 Jalan Tan Tock Seng, Singapore, 308433 Singapore; 3 The Palliative Care Centre for Excellence in Research and Education, Dover Park Hospice, 10 Jalan Tan Tock Seng, Singapore, 308436 Singapore

**Keywords:** Abstract, Classification, Semi-automation

## Abstract

We demonstrate the performance and workload impact of incorporating a natural language model, pretrained on citations of biomedical literature, on a workflow of abstract screening for studies on prognostic factors in end-stage lung disease. The model was optimized on one-third of the abstracts, and model performance on the remaining abstracts was reported. Performance of the model, in terms of sensitivity, precision, F1 and inter-rater agreement, was moderate in comparison with other published models. However, incorporating it into the screening workflow, with the second reviewer screening only abstracts with conflicting decisions, translated into a 65% reduction in the number of abstracts screened by the second reviewer. Subsequent work will look at incorporating the pre-trained BERT model into screening workflows for other studies prospectively, as well as improving model performance.

**Supplementary Information:**

The online version contains supplementary material available at 10.1186/s13643-023-02353-8.

## Introduction

In recent years, there has been a growth in interest in using artificial intelligence methods in systematic reviews (SRs) [1], in particular for the stage of literature screening [2]. As the number of titles and abstracts to be screened for suitability for inclusion in a review often involves numerous hours of repetitive work, semi-automation of this stage has been suggested to deliver workload and time savings with acceptable recall and precision [3–5].

One approach targets the automated classification of studies for inclusion using prediction models. In recent work, Aum et al. developed a Bidirectional Encoder Representations from Transformer (BERT) algorithm that was pretrained on published SRs and fine-tuned on another SR, with good classification performance. The authors recommended generalizing the use of BERT-based models for this purpose, by pre-training with information from a particular clinical domain and optimizing the predictions for the individual review only at the last fine-tuning step [6]. In this letter, we demonstrate the performance and workload impact of incorporating a BERT model pretrained on citations of biomedical literature in our own abstract screening workflow.

## Methods

We used abstracts retrieved from a previous literature search on prognostic factors in end-stage lung disease [7]. Bibliographic databases such as MEDLINE, Embase, PubMed, CINAHL, Cochrane Library and Web of Science were searched using a pre-defined search strategy and inclusion criteria (Additional file 1). A total of 21,645 abstracts were retrieved, and based on screening by reviewers, 530 (2.5%) of the studies were included in the subsequent stage, where the full text of the articles was retrieved for thorough reading.

The dataset of 21,645 abstracts consisted of the text within the abstract, excluding the title, as well as an indication of whether the abstract was classified as included by the human reviewers. For model validation, the dataset was split into a training set of 7142 abstracts (33%), and a test set of 14,503 abstracts (67%). We then used the training set to fine-tune a BERT model pretrained on citations from MEDLINE/PubMed (pBERT). A batch size of 64 was used, and convergence over 100 epochs was assessed [8].

We then applied the fine-tuned pBERT to the test set and labelled 2.5% of articles with the highest predicted probabilities of inclusion as included in the review by pBERT. Based on this set of labels, we assessed sensitivity, precision, F1 and accuracy of pBERT, as well as the proportion of conflicts and level of inter-rater agreement, which was measured by Cohen’s kappa (Table 1). We also report the reduction in workload in a hypothetical scenario where pBERT performs screening as the second reviewer for 67% of the articles.

**Table 1:** List of performance measures assessed

Measure	Definition	Estimate
Recall/sensitivity	$\frac{\mathrm{Number}\;\mathrm{of}\;\mathrm{abstracts}\;\mathrm{included}\;\mathrm{by}\;\mathrm{human}\;\mathrm{reviewer}\;\mathrm{and}\;\mathrm{pBERT}}{Nu\mathrm{mber}\;\mathrm{of}\;\mathrm{abstracts}\;\mathrm{included}\;\mathrm{by}\;\mathrm{human}\;\mathrm{reviewer}}$	37.7%
Precision/positive predictive value	$\frac{\mathrm{Number}\;\mathrm{of}\;\mathrm{abstracts}\;\mathrm{included}\;\mathrm{by}\;\mathrm{human}\;\mathrm{reviewer}\;\mathrm{and}\;\mathrm{pBERT}}{Nu\mathrm{mber}\;\mathrm{of}\;\mathrm{abstracts}\;\mathrm{included}\;\mathrm{by}\;\mathrm{pBERT}}$	37.7%
F1	$2 \times \frac{precision \times recall }{precision+recall}$	37.7%
Accuracy	$\frac{\mathrm{Number}\;\mathrm{of}\;\mathrm{abstracts}\;\mathrm{included}\;\mathrm{by}\;\mathrm{human}\;\mathrm{reviewer}\;\mathrm{and}\;\mathrm{pBERT}}{\mathrm{Total}\;\mathrm{number}\;\mathrm{of}\;\mathrm{abstracts}\;\mathrm{screened}}$	70.2%
Disagreement	$\frac{\mathrm{Number}\;\mathrm{of}\;\mathrm{abstracts}\;\mathrm{with}\;\mathrm{different}\;\mathrm{decisions}\;\mathrm{by}\;\mathrm{human}\;\mathrm{reviewer}\;\mathrm{and}\;\mathrm{pBERT}}{\mathrm{Total}\;\mathrm{number}\;\mathrm{of}\;\mathrm{abstracts}\;\mathrm{screened}}$	3.0%

## Results

Of the 14,503 abstracts in the test set, the human reviewers deemed 355 (2.5%) to be relevant and suitable for inclusion in the subsequent stage of review. Sensitivity, precision and F1 of pBERT were 37.7%, while disagreement occurred for 3.0% of all articles screened. Cohen’s Kappa was 0.70, indicating moderate agreement between the reviewers and pBERT (Table 1).

In the traditional screening process, each of the two human reviewers would have to screen all 21,645 articles for relevance to the study, before reviewing any conflicts in their decisions. With pBERT incorporated into the screening workflow, both reviewers would screen the first 33% of articles, and pBERT would be fine-tuned based on this training set.

For the remaining 14,503 articles, the first reviewer (R1) would screen all the articles in accordance with the traditional workflow. pBERT would then replace the second human reviewer (R2) in identifying studies for inclusion, while R2 would only step in to review articles with conflicting decisions. In this scenario, R1 and pBERT would have agreed on decisions for 14,061 articles, leaving 442 articles (3% of 14,503) for R2 to review. Hence, R2 would have to review only 7,584 articles (7,142 + 442) or 35% of the original 21,645 articles.

## Discussion

We applied a BERT model pretrained on biomedical literature to our data, with moderate model performance. Having a sensitivity of 37.7% entails that pBERT can only be used as an assistant alongside a human reviewer to increase the efficiency of screening, as opposed to being a standalone tool for automation of screening. While pBERT did not perform as well in terms of traditional metrics compared to recent models [6, 9], our dataset did have a lower inclusion rate of 2.5%, compared to 11% and 19% in both studies, impacting the predictive ability of the model.

Nonetheless, despite the constrained performance of pBERT, we were able to demonstrate that incorporating pBERT in our workflow would have reduced the workload of a second human reviewer to a third of the initial volume. Our results suggest that involving predictive tools to screen out irrelevant articles, which often comprise the bulk of the abstracts, can improve efficiency of screening processes in comparison to traditional approaches. However, while there is interest to fully automate the task of screening without human intervention, we emphasize that the role of a human reviewer remains pertinent to ensure all potentially relevant articles are included in the study [10].

## Conclusion

For semi-automation of screening of literature on prognostic factors in end-stage lung disease, we used a BERT model trained on biomedical literature to identify abstracts that were relevant to the topic and demonstrated a substantial reduction in screening workload. Subsequent work will look at integrating the current version of pBERT into screening workflows for other studies prospectively, as well as incorporating other ensemble methods to develop models with improved sensitivity to identify abstracts of relevance to the research question.

### Supplementary Information

**Additional file 1.** Search criteria and strategy. **Appendix 1.** Study eligibility criteria. **Appendix 2.** Search strategy.

## Data Availability

Data arising from the review may be made available from the corresponding author upon reasonable request.

## Abbreviations

AI: Artificial intelligence
BERT: Bidirectional Encoder Representations from Transformer
pBERT: PubMed BERT
R1: Reviewer 1
R2: Reviewer 2
SR: Systematic reviews
