# Supplementary material for: Semi-automating abstract screening with a natural language model pretrained on biomedical literature
Source: Syst Rev. 2023 Sep 23;12:172. doi: 10.1186/s13643-023-02353-8 (PMC10517490; doi:10.1186/s13643-023-02353-8)
Supplement: Supplementary file 1 — Additional file 1. Search criteria and strategy. Appendix 1. Study eligibility criteria. Appendix 2. Search strategy. [file 13643_2023_2353_MOESM1_ESM.docx]

Additional File 1. Search criteria and strategy

Appendix 1. Study eligibility criteria

| **Study Characteristics** | **Inclusion Criteria** | **Exclusion Criteria** |
| --- | --- | --- |
| Type of publication | - Peer-reviewed journal articles, PhD Thesis, grey literature | - Editorials, letters, commentaries, abstracts with insufficient information, reviews, genetic studies based on or including animals |
| Study population | Populations with chronic lung disease, including but not restricted to:   - Interstitial lung disease - Pulmonary hypertension associated with chronic lung disease (Group 3) - Bronchiectasis | Patients who:   - were diagnosed with COPD or pneumonia - were diagnosed with acute respiratory failure or lung injury - were diagnosed with non-group 3 pulmonary hypertension - have co-occurring malignancies/HIV - underwent lung transplant or other procedures   Populations containing excluded patient subgroups (e.g. COPD, cancer) |
| Study design | - Quantitative studies which employed prospective, retrospective, longitudinal, RCT, and cross-sectional designs - Studies that performed external validation and reported variables or referenced the primary study | - Qualitative studies - Cost-effectiveness studies |
| Intervention | - Treatment or intervention studies | - N.A. |
| Analysis | - Studies that conducted multivariable analysis - Studies that analyzed any factors associated with or predicted mortality/survival - Studies reporting subgroup-specific multivariate analysis for populations with only one subtype of interest | - Studies that conducted univariable analysis only - Studies that conducted competing risk analyses |
| Outcome | - All-cause mortality within 3 years   - For studies with no defined mortality risk period, maximum follow-up time must fall within 3 years   - If studies did not report maximum follow-up time, mean + 3 standard deviations, or median + 2 interquartile range must fall within 3 years | - Composite outcomes (e.g. death and hospitalization) - Only non-mortality outcomes assessed |
| Language | - English | - Other languages |
| Geography | - Worldwide | - N.A. |

Appendix 2. Search strategy

| Database | Search strategy |
| --- | --- |
| Medline | Database: Ovid MEDLINE(R) and Epub Ahead of Print, In-Process & Other Non-Indexed Citations, Daily and Versions(R) <1946 to October 16, 2020>  Search Strategy:  --------------------------------------------------------------------------------  1 exp Bronchiectasis/ (9274)  2 lung diseases/ or hypertension, pulmonary/ or pulmonary arterial hypertension/ or lung abscess/ or lung diseases, interstitial/ or alveolitis, extrinsic allergic/ or bird fancier's lung/ or farmer's lung/ or silo filler's disease/ or trichosporonosis/ or idiopathic interstitial pneumonias/ or cryptogenic organizing pneumonia/ or pneumoconiosis/ or anthracosis/ or anthracosilicosis/ or asbestosis/ or berylliosis/ or byssinosis/ or caplan syndrome/ or siderosis/ or silicosis/ or silicotuberculosis/ or lung, hyperlucent/ or pulmonary fibrosis/ or idiopathic pulmonary fibrosis/ (157207)  3 exp Connective Tissue Diseases/ (306179)  4 lung diseases, interstitial/ or alveolitis, extrinsic allergic.mp. [mp=title, abstract, original title, name of substance word, subject heading word, floating sub-heading word, keyword heading word, organism supplementary concept word, protocol supplementary concept word, rare disease supplementary concept word, unique identifier, synonyms] (12360)  5 3 and 4 (2411)  6 (((Disease, pulmonary or diseases, pulmonary or pulmonary diseas* or advanced pulmonary or advanced lung diseases or end stage?) adj3 lung diseas*) or hypertension pulmonary or lung diseas*, interstitial lung diseas*or pulmonary fibrosis or late stage lung disease or Idiopathic interstitial pneumonia or Chronic respiratory failure* or secondary pulmonary hypertension or Cor pulmonale or Bronchiectasis).ab,ti,tw. (17590)  7 1 or 2 or 5 or 6 (173531)  8 survival analysis/ or kaplan-meier estimate/ or proportional hazards models/ or Survival Rate/ or Life Expectancy/ or mortality/ or fatal outcome/ or hospital mortality/ or prognosis/ or exp medical futility/ or exp nomograms/ or Logistic Models/ (1066559)  9 ("death rate?" or mortality or "survival rate?" or "death risk?" or "life expectancy" or "survival analysis" or survival or prognosis or "prognostic factor?" or prediction? or "prognostic model?" or "prediction model?" or "survival model?" or death or dying or "logistic model?" or "logistic regression" or "logit model?").ab,ti,tw. (2872905)  10 8 or 9 (3277970)  11 Epidemiologic studies/ or exp case control studies/ or exp cohort studies/ or Case control.tw. or (cohort adj (study or studies)).tw. or Cohort analy$.tw. or (Follow up adj (study or studies)).tw. or (observational adj (study or studies)).tw. or Longitudinal.tw. or Retrospective.tw. or Cross sectional.tw. or Cross-sectional studies/ (3076087)  12 Randomized controlled trials as Topic/ or Randomized controlled trial/ or Random allocation/ or Double blind method/ or Single blind method/ or Clinical trial/ or exp Clinical Trials as Topic/ (1187317)  13 ((clinic$ adj trial$1) or ((singl$ or doubl$ or treb$ or tripl$) adj (blind$3 or mask$3))).tw. or Placebos/ or Placebo$.tw. or Randomly allocated.tw. or (allocated adj2 random).tw. (646442)  14 11 or 12 or 13 (4234640)  15 15 Case report.tw. or Letter/ or Historical article/ or Review of reported cases.pt. or Review, multicase.pt. (1456840)  16 14 not 15 (4150110)  17 7 and 10 and 16 (11432)  18 limit 17 to (english language and humans and yr="2000 - 2020" and "all adult (19 plus years)") (6448) |
| EMBASE | 1. exp bronchiectasis/ (20625) 2. lung disease/ or pulmonary hypertension/ or lung abscess/ or interstitial lung disease/ or allergic pneumonitis/ or bird breeder lung/ or occupational lung disease/ or trichosporonosis/ or interstitial pneumonia/ or bronchiolitis obliterans organizing pneumonia/ or pneumoconiosis/ or anthracosis/ or asbestosis/ or berylliosis/ or byssinosis/ or siderosis/ or silicosis/ or lung tuberculosis/ or hypertranslucent lung/ or lung fibrosis/ or fibrosing alveolitis/ (301494) 3. exp connective tissue disease/ (438688) 4. interstitial lung disease/ or allergic pneumonitis/ (27668) 5. (((Disease, pulmonary or diseases, pulmonary or pulmonary diseas* or advanced pulmonary or advanced lung diseases or end stage?) adj3 lung diseas*) or hypertension pulmonary or lung diseas*, interstitial lung diseas*or pulmonary fibrosis or late stage lung disease or Idiopathic interstitial pneumonia or Chronic respiratory failure* or secondary pulmonary hypertension or Cor pulmonale or Bronchiectasis).ab,ti,tw. (25688) 6. 3 and 4 (8347) 7. 1 or 2 or 5 or 6 (322813) 8. survival analysis/ or kaplan meier method/ or proportional hazards model/ or survival rate/ or life expectancy/ or mortality/ or fatality/ or hospital mortality/ or prognosis/ or exp treatment outcome/ or nomogram/ or statistical model.mp. [mp=title, abstract, heading word, drug trade name, original title, device manufacturer, drug manufacturer, device trade name, keyword, floating subheading word, candidate term word] (3279089) 9. ("death rate?" or mortality or "survival rate?" or "death risk?" or "life expectancy" or "survival analysis" or survival or prognosis or "prognostic factor?" or prediction? or "prognostic model?" or "prediction model?" or "survival model?" or death or dying or "logistic model?" or "logistic regression" or "logit model?").ab,ti,tw. (4043775) 10. 8 or 9 (5791882) 11. "randomized controlled trial (topic)"/ or randomized controlled trial/ or randomization/ or double blind procedure/ or single blind procedure/ or clinical trial/ or exp "clinical trial (topic)"/ (1724593) 12. ((clinic$ adj trial$1) or ((singl$ or doubl$ or treb$ or tripl$) adj (blind$3 or mask$3))).tw. or placebo/ or Placebo$.tw. or Randomly allocated.tw. or (allocated adj2 random).tw. (1027218) 13. epidemiology/ or exp case control study/ or cohort analysis/ or cross-sectional study/ or (Case control or (cohort adj (study or studies)) or Cohort analy$ or (Follow up adj (study or studies)) or (observational adj (study or studies)) or Longitudinal or Retrospective or Cross sectional).tw. (2736047) 14. 11 or 12 or 13 (4674142) 15. Case report.tw. or letter/ or Review of reported cases.pt. or Review, multicase.pt. (1498683) 16. 14 not 15 (4595846) 17. 7 and 10 and 16 (32383) 18. limit 17 to (human and english language and exclude medline journals and yr="2000 - 2020" and (adult <18 to 64 years> or aged <65+ years>)) (1537) |
| PubMed | 1. "bronchiectasis"[MeSH Terms] 2. "lung diseases"[MeSH Terms:noexp] OR "hypertension, pulmonary"[MeSH Terms:noexp] OR "pulmonary arterial hypertension"[MeSH Terms:noexp] OR "lung abscess"[MeSH Terms:noexp] OR "lung diseases, interstitial"[MeSH Terms:noexp] OR "alveolitis, extrinsic allergic"[MeSH Terms:noexp] OR "bird fancier s lung"[MeSH Terms:noexp] OR "farmer s lung"[MeSH Terms:noexp] OR "silo filler s disease"[MeSH Terms:noexp] OR "trichosporonosis"[MeSH Terms:noexp] OR "idiopathic interstitial pneumonias"[MeSH Terms:noexp] OR "cryptogenic organizing pneumonia"[MeSH Terms:noexp] OR "pneumoconiosis"[MeSH Terms:noexp] OR "anthracosis"[MeSH Terms:noexp] OR "anthracosilicosis"[MeSH Terms:noexp] OR "asbestosis"[MeSH Terms:noexp] OR "berylliosis"[MeSH Terms:noexp] OR "byssinosis"[MeSH Terms:noexp] OR "caplan syndrome"[MeSH Terms:noexp] OR "siderosis"[MeSH Terms:noexp] OR "silicosis"[MeSH Terms:noexp] OR "silicotuberculosis"[MeSH Terms:noexp] OR "lung, hyperlucent"[MeSH Terms:noexp] OR "pulmonary fibrosis"[MeSH Terms:noexp] OR "idiopathic pulmonary fibrosis"[MeSH Terms:noexp 3. "connective tissue diseases"[MeSH Terms] 4. "lung diseases, interstitial"[MeSH Terms:noexp] OR "alveolitis, extrinsic allergic"[MeSH Terms:noexp] 5. #3 and #4 6. "disease pulmonary"[Title/Abstract] OR "diseases pulmonary"[Title/Abstract] OR "pulmonary disease"[Title/Abstract] OR "advanced pulmonary"[Title/Abstract] OR "advanced lung diseases"[Title/Abstract] OR "end stage lung disease"[Title/Abstract] OR "hypertension pulmonary"[Title/Abstract] OR (("lung"[MeSH Terms] OR "lung"[All Fields]) AND "disease interstitial lung disease"[Title/Abstract]) OR "pulmonary fibrosis"[Title/Abstract] OR ("late"[All Fields] AND "stage lung disease"[Title/Abstract]) OR "idiopathic interstitial pneumonia"[Title/Abstract] OR "chronic respiratory failure"[Title/Abstract] OR "secondary pulmonary hypertension"[Title/Abstract] OR "cor pulmonale"[Title/Abstract] OR "Bronchiectasis"[Title/Abstract] 7. #1 or #2 or #5 or #6 8. "survival analysis"[MeSH Terms:noexp] OR "Kaplan-Meier Estimate"[MeSH Terms:noexp] OR "proportional hazards models"[MeSH Terms:noexp] OR "survival rate"[MeSH Terms:noexp] OR "life expectancy"[MeSH Terms:noexp] OR "mortality"[MeSH Terms:noexp] OR "fatal outcome"[MeSH Terms:noexp] OR "hospital mortality"[MeSH Terms:noexp] OR "prognosis"[MeSH Terms:noexp] OR "medical futility"[MeSH Terms] OR "nomograms"[MeSH Terms] OR "logistic models"[MeSH Terms:noexp] OR "death rate"[Title/Abstract] OR "death rates"[Title/Abstract] OR "mortality"[Title/Abstract] OR "survival rate"[Title/Abstract] OR "survival rates"[Title/Abstract] OR "death risk"[Title/Abstract] OR "death risks"[Title/Abstract] OR "life expectancy"[Title/Abstract] OR "survival analysis"[Title/Abstract] OR "survival"[Title/Abstract] OR "prognosis"[Title/Abstract] OR "prognostic factor"[Title/Abstract] OR "prognostic factors"[Title/Abstract] OR "prediction"[Title/Abstract] OR "predictions"[Title/Abstract] OR "prognostic model"[Title/Abstract] OR "prognostic models"[Title/Abstract] OR "prediction model"[Title/Abstract] OR "prediction models"[Title/Abstract] OR "survival model"[Title/Abstract] OR "survival models"[Title/Abstract] OR "death"[Title/Abstract] OR "dying"[Title/Abstract] OR "logistic model"[Title/Abstract] OR "logistic models"[Title/Abstract] OR "logistic regression"[Title/Abstract] OR "logit model"[Title/Abstract] OR "logit models"[Title/Abstract] 9. "death rate"[Title/Abstract] OR "death rates"[Title/Abstract] OR "mortality"[Title/Abstract] OR "survival rate"[Title/Abstract] OR "survival rates"[Title/Abstract] OR "death risk"[Title/Abstract] OR "death risks"[Title/Abstract] OR "life expectancy"[Title/Abstract] OR "survival analysis"[Title/Abstract] OR "survival"[Title/Abstract] OR "prognosis"[Title/Abstract] OR "prognostic factor"[Title/Abstract] OR "prognostic factors"[Title/Abstract] OR "prediction"[Title/Abstract] OR "predictions"[Title/Abstract] OR "prognostic model"[Title/Abstract] OR "prognostic models"[Title/Abstract] OR "prediction model"[Title/Abstract] OR "prediction models"[Title/Abstract] OR "survival model"[Title/Abstract] OR "survival models"[Title/Abstract] OR "death"[Title/Abstract] OR "dying"[Title/Abstract] OR "logistic model"[Title/Abstract] OR "logistic models"[Title/Abstract] OR "logistic regression"[Title/Abstract] OR "logit model"[Title/Abstract] OR "logit models"[Title/Abstract] 10. #8 or #9 11. "epidemiologic studies"[MeSH Terms:noexp] OR "case control studies"[MeSH Terms] OR "cohort studies"[MeSH Terms] OR "case control"[Text Word] OR "cohort study"[Text Word] OR "cohort studies"[Text Word] OR "cohort analy*"[Text Word] OR "Follow up study"[Text Word] OR "Follow up studies"[Text Word] OR "observational study"[Text Word] OR "observational studies"[Text Word] OR "Longitudinal"[Text Word] OR "Retrospective"[Text Word] OR "Cross sectional"[Text Word] OR "cross sectional studies"[MeSH Terms:noexp] 12. "randomized controlled trials as topic"[MeSH Terms:noexp] OR "random allocation"[MeSH Terms:noexp] OR "double blind method"[MeSH Terms:noexp] OR "single blind method"[MeSH Terms:noexp] OR "clinical trials as topic"[MeSH Terms] 13. "placebo*"[Text Word] OR "randomly allocated"[Text Word] OR "placebos"[MeSH Terms:noexp] 14. #11 or #12 or #13 15. "case report"[Text Word] 16. #15 or #14 17. #14 not #15 18. #7 and #10 and #17 |
| CINAHL | S1) MH Bronchiectasis  S2) MH lung diseases or hypertension, pulmonary or pulmonary arterial hypertension or lung abscess or lung diseases, interstitial or alveolitis, extrinsic allergic or bird fancier's lung or farmer's lung or silo filler's disease or trichosporonosis or idiopathic interstitial pneumonias or cryptogenic organizing pneumonia or pneumoconiosis or anthracosis or anthracosilicosis or asbestosis or berylliosis or byssinosis or caplan syndrome or siderosis or silicosis or silicotuberculosis or lung, hyperlucent or pulmonary fibrosis or idiopathic pulmonary fibrosis  S3) MH Connective Tissue Diseases  S4) lung diseases, interstitial or alveolitis, extrinsic allergic  S5) S3 AND S4  S6) TI Disease, pulmonary or diseases, pulmonary or pulmonary diseas* or advanced pulmonary or advanced lung diseases or end stage?) adj3 lung diseas*) or hypertension pulmonary or lung diseas*, interstitial lung diseas*or pulmonary fibrosis or late stage lung disease or Idiopathic interstitial pneumonia or Chronic respiratory failure* or secondary pulmonary hypertension or Cor pulmonale or Bronchiectasis  S7) S1 OR S2 OR S5 OR S6  S8) (MH "Survival Analysis") OR (MH "Kaplan-Meier Estimator") OR (MH "Cox Proportional Hazards Model") OR (MH "Life Expectancy") OR (MH "Hospital Mortality") OR (MH "Mortality") OR (MH "Fatal Outcome") OR (MH "Prognosis") OR (MH "Medical Futility")  S9) TI ( "death rate#" or mortality or "survival rate#" or "death risk#" or" life expectancy" or "survival analysis" or survival or prognosis or "prognostic factor#" or prediction# or "prognostic model#" or "prediction model#" or "survival model#" or death or dying or "logistic model#" or "logistic regression" or "logit model#" ) OR AB("death rate#" or mortality or "survival rate#" or "death risk#" or "life expectancy" or "survival analysis" or survival or prognosis or "prognostic factor#" or prediction# or "prognostic model#" or "prediction model#" or "survival model#" or death or dying or "logistic model#" or "logistic regression" or "logit model#" )  S10) S8 OR S9  S11) (MH "Epidemiological Research") OR (MH "Case Control Studies+")OR (MH "Prospective Studies+") OR TI "Case control" OR AB "Case control" OR TI ( cohort N (study or studies) ) ORAB ( cohort N (study or studies) ) OR TI "Cohort analy*" OR AB "Cohort analy*" OR TI ( ("Followup" N (study or studies))or(observational N (study or studies)) or Longitudinal or Retrospective or "Cross sectional" ) OR AB(("Follow up" N (study or studies)) or(observational N (study or studies)) or Longitudinal or Retrospective or "Cross sectional" ) OR (MH "Cross Sectional Studies")  S12) (MH "Randomized Controlled Trials") OR (MH "Random Assignment") OR (MH "Double-Blind Studies") OR (MH "Single-Blind Studies") OR (MH "Clinical Trials")  S13) TI ( ((clinic* N trial*1)or ((singl* or doubl* or treb* or tripl*) N (blind*3 or mask*3))) or Placebo* or "Randomly allocated" or(allocated N2 random) )OR AB ( ((clinic* N trial*1)or ((singl* or doubl*or treb* or tripl*) N(blind*3or mask*3))) or Placebo*or "Randomly allocated" or (allocated N2random)) OR (MH "Placebos")  S14) S11 OR S12 OR S13  S15) TI "Case report" OR AB "Case report" OR PT "Review of reported cases" OR PT "Review, multicase"  S16) 14 not 15  S17) (14 not 15) AND (S7 AND S10 AND S16) |
| Cochrane | #1 MeSH descriptor: [Bronchiectasis] explode all trees 333  #2 MeSH descriptor: [Lung Diseases] this term only 3064  #3 MeSH descriptor: [Hypertension, Pulmonary] this term only 949  #4 MeSH descriptor: [Pulmonary Arterial Hypertension] this term only 10  #5 MeSH descriptor: [Lung Abscess] this term only 22  #6 MeSH descriptor: [Lung Diseases, Interstitial] this term only 232  #7 MeSH descriptor: [Alveolitis, Extrinsic Allergic] this term only 14  #8 MeSH descriptor: [Bird Fancier's Lung] this term only 3  #9 MeSH descriptor: [Farmer's Lung] this term only 8  #10 MeSH descriptor: [Silo Filler's Disease] this term only 0  #11 MeSH descriptor: [Trichosporonosis] this term only 0  #12 MeSH descriptor: [Idiopathic Interstitial Pneumonias] this term only 173  #13 MeSH descriptor: [Cryptogenic Organizing Pneumonia] this term only 4  #14 MeSH descriptor: [Pneumoconiosis] this term only 32  #15 MeSH descriptor: [Anthracosis] this term only 3  #16 MeSH descriptor: [Anthracosilicosis] this term only 2  #17 MeSH descriptor: [Asbestosis] this term only 13  #18 MeSH descriptor: [Berylliosis] this term only 6  #19 MeSH descriptor: [Byssinosis] this term only 7  #20 MeSH descriptor: [Caplan Syndrome] this term only 0  #21 MeSH descriptor: [Siderosis] this term only 5  #22 MeSH descriptor: [Silicosis] this term only 31  #23 MeSH descriptor: [Silicotuberculosis] this term only 4  #24 MeSH descriptor: [Lung, Hyperlucent] explode all trees 1  #25 MeSH descriptor: [Pulmonary Fibrosis] this term only 404  #26 MeSH descriptor: [Idiopathic Pulmonary Fibrosis] this term only 307  #27 MeSH descriptor: [Connective Tissue Diseases] explode all trees 9561  #28 MeSH descriptor: [Lung Diseases, Interstitial] this term only 232  #29 MeSH descriptor: [Alveolitis, Extrinsic Allergic] this term only 14  #30 #28 or #29 244  #31 #27 and #30 58  #32 (Disease, pulmonary or diseases, pulmonary or pulmonary diseas* or advanced pulmonary or advanced lung diseases or end stage? adj3 lung diseas* or hypertension pulmonary or lung diseas*, interstitial lung diseas*or pulmonary fibrosis or late stage lung disease or Idiopathic interstitial pneumonia or Chronic respiratory failure* or secondary pulmonary hypertension or Cor pulmonale or Bronchiectasis):ti,ab,kw 34254  #33 #1 or #2 or #3 or #4 or #5 or #6 or #7 or #8 or #9 or #10 or #11 or #11 or #12 or #13 or #14 or #15 or #16 or #17 or #18 or #19 or #20 or #21 or #22 or #23 or #24 or #25 or #26 or #31 5013  #34 MeSH descriptor: [Survival Analysis] this term only 8129  #35 MeSH descriptor: [Kaplan-Meier Estimate] this term only 4959  #36 MeSH descriptor: [Proportional Hazards Models] this term only 5016  #37 MeSH descriptor: [Survival Rate] this term only 9886  #38 MeSH descriptor: [Mortality] this term only 550  #39 MeSH descriptor: [Fatal Outcome] this term only 12  #40 MeSH descriptor: [Hospital Mortality] this term only 1156  #41 MeSH descriptor: [Prognosis] this term only 13396  #42 MeSH descriptor: [Medical Futility] this term only 36  #43 MeSH descriptor: [Nomograms] this term only 72  #44 MeSH descriptor: [Logistic Models] this term only 5115  #45 ("death rate?" or mortality or "survival rate?" or "death risk?" or "life expectancy" or "survival analysis" or survival or prognosis or "prognostic factor?" or prediction? or "prognostic model?" or "prediction model?" or "survival model?" or death or dying or "logistic model?" or "logistic regression" or "logit model?"):ti,ab,kw 231923  #46 #34 or #35 or #36 or #37 or #38 or #39 or #40 or #41 or #42 or #43 or #44 or #45 236280  #47 MeSH descriptor: [Epidemiologic Studies] this term only 41  #48 MeSH descriptor: [Case-Control Studies] explode all trees 12637  #49 MeSH descriptor: [Cohort Studies] explode all trees 147464  #50 MeSH descriptor: [Cross-Sectional Studies] this term only 4292  #51 ("Case control" or "cohort study" OR "cohort studies" or "Cohort analy*" or "Follow up study" OR "follow up studies" or "observational study" OR "observational studies" or Longitudinal or Retrospective or "Cross sectional"):ti,ab,kw 140738  #52 MeSH descriptor: [Randomized Controlled Trials as Topic] this term only 12396  #53 MeSH descriptor: [Randomized Controlled Trial] this term only 118  #54 MeSH descriptor: [Random Allocation] this term only 20604  #55 MeSH descriptor: [Double-Blind Method] this term only 137713  #56 MeSH descriptor: [Single-Blind Method] this term only 20810  #57 MeSH descriptor: [Clinical Trial] this term only 27  #58 MeSH descriptor: [Clinical Trials as Topic] explode all trees 47906  #59 MeSH descriptor: [Placebos] this term only 24036  #60 ((((clinic* NEAR trial*1) or ((singl* or doubl* or treb* or tripl*) NEAR (blind*3 or mask*3))) or Placebo* or "Randomly allocated" or (allocated NEAR/2 random))):ti,ab,kw 339747  #61 #47 or #48 or #49 or #50 or #51 or #52 or #53 or #54 or #55 or #56 or #57 or #58 or #59 or #60 582502  #62 MeSH descriptor: [Letter] this term only 0  #63 (case report*):ti,ab,kw 18852  #64 MeSH descriptor: [Historical Article] this term only 0  #65 (review of historical case*):pt 0  #66 ((Review, multicase)):pt 0  #67 #62 or #63 or #64 or #65 or #66 18852  #68 #61 not #67 574285   1. #69 #33 and #46 and #68 628 |
| Web of Science | 1. TS=(Bronchiectasis) 2. TS=("lung diseases" or "hypertension, pulmonary" or "pulmonary arterial hypertension" or "lung abscess" or "lung diseases, interstitial" or "alveolitis, extrinsic allergic" or "bird fancier's lung" or "farmer's lung" or "silo filler's disease" or trichosporonosis or "idiopathic interstitial pneumonias" or "cryptogenic organizing pneumonia" or pneumoconiosis or anthracosis or anthracosilicosis or asbestosis or berylliosis or byssinosis or "caplan syndrome" or siderosis or silicosis or silicotuberculosis or "lung, hyperlucent" or "pulmonary fibrosis" or "idiopathic pulmonary fibrosis") 3. TS=("Disease, pulmonary" or "diseases, pulmonary" or "pulmonary diseas*" or "advanced pulmonary" or "advanced lung diseases" or "end stage lung diseas*" or "hypertension pulmonary" or "lung diseas*"or "interstitial lung diseas*" or "pulmonary fibrosis" or "late stage lung disease" or "Idiopathic interstitial pneumonia" or "Chronic respiratory failure*" or "secondary pulmonary hypertension" or "Cor pulmonale" or Bronchiectasis) 4. TS=("Connective Tissue Diseases") 5. TS=("lung diseases, interstitial" or "alveolitis, extrinsic allergic") 6. #5 AND #4 7. #6 OR #3 OR #2 OR #1 8. TS=("death rate$" or mortality or "survival rate$" or "death risk$" or "life expectancy" or "survival analysis" or survival or prognosis or "prognostic factor$" or prediction$ or "prognostic model$" or "prediction model$" or "survival model$" or death or dying or "logistic model$" or "logistic regression" or "logit model$") 9. TS=("Case control" or (cohort NEAR (study or studies) ) or Cohort analy* or ("Follow up" NEAR (study or studies) ) or (observational NEAR (study or studies) ) or Longitudinal or Retrospective or "Cross sectional") 10. TS=(((clinic* NEAR trial*1) or ((singl* or doubl* or treb* or tripl*) NEAR (blind*3 or mask*3) )) or Placebo* or "Randomly allocated" or (allocated NEAR/2 random) ) 11. #10 OR #9 12. TS=("Case report") 13. #11 not #12 14. #13 AND #8 AND #7 15. (#13 AND #8 AND #7) AND LANGUAGE: (English) 16. 16. Refined by: PUBLICATION YEARS: ( 2020 OR 2012 OR 2004 OR 2019 OR 2011 OR 2003 OR 2018 OR 2010 OR 2002 OR 2017 OR 2009 OR 2001 OR 2016 OR 2008 OR 2000OR 2015 OR 2007 OR 2014 OR 2006 OR 2013 OR 2005 ) |
